# Characterization of a Novel Polerovirus Infecting Maize in China

**DOI:** 10.3390/v8050120

**Published:** 2016-04-28

**Authors:** Sha Chen, Guangzhuang Jiang, Jianxiang Wu, Yong Liu, Yajuan Qian, Xueping Zhou

**Affiliations:** 1 Institute of Biotechnology, Zhejiang University, Hangzhou 310058, China; sophiachen920@126.com (S.C.); jgzzero@163.com (G.J.); wujx@zju.edu.cn (J.W.); 2 State Key Laboratory for Biology of Plant Diseases and Insect Pests, Institute of Plant Protection, Chinese Academy of Agricultural Sciences, Beijing 100193, China; 3 Key Laboratory of Pest Management of Horticultural Crop of Hunan Province, Hunan Plant Protection Institute, Hunan Academy of Agricultural Science, Changsha 410125, China; haoasliu@163.com

**Keywords:** Maize yellow mosaic virus, deep sequencing, infectious clone, suppressor, molecular variation, sRNA

## Abstract

A novel virus, tentatively named Maize Yellow Mosaic Virus (MaYMV), was identified from the field-grown maize plants showing yellow mosaic symptoms on the leaves collected from the Yunnan Province of China by the deep sequencing of small RNAs. The complete 5642 nucleotide (nt)-long genome of the MaYMV shared the highest nucleotide sequence identity (73%) to Maize Yellow Dwarf Virus-RMV. Sequence comparisons and phylogenetic analyses suggested that MaYMV represents a new member of the genus *Polerovirus* in the family *Luteoviridae*. Furthermore, the P0 protein encoded by MaYMV was demonstrated to inhibit both local and systemic RNA silencing by co-infiltration assays using transgenic *Nicotiana benthamiana* line 16c carrying the GFP reporter gene, which further supported the identification of a new polerovirus. The biologically-active cDNA clone of MaYMV was generated by inserting the full-length cDNA of MaYMV into the binary vector pCB301. RT-PCR and Northern blot analyses showed that this clone was systemically infectious upon agro-inoculation into *N. benthamiana*. Subsequently, 13 different isolates of MaYMV from field-grown maize plants in different geographical locations of Yunnan and Guizhou provinces of China were sequenced. Analyses of their molecular variation indicate that the 3′ half of P3–P5 read-through protein coding region was the most variable, whereas the coat protein- (CP-) and movement protein- (MP-)coding regions were the most conserved.

## 1. Introduction

The family *Luteoviridae* belongs to the picornavirus-like superfamily of positive-strand RNA viruses [1] and currently includes three genera (*Luteovirus*, *Polerovirus*, and *Enamovirus*). The plant viruses in the genus *Polerovirus* possess monopartite RNA genomes of about 5–6 kb in length [2] that typically contain six open reading frames (ORF) referred to as ORF0 to ORF5, and three untranslated regions (UTRs), including the 5′ UTR, the 3′ UTR, and the intergenic UTR between ORF2 and ORF3 [3,4,5,6]. The protein encoded by ORF0 has been shown to function as an RNA-silencing suppressor (RSS) [7,8,9], which is distinct from the members of genus *Luteovirus* that lack ORF0 [10]. The ORF1 overlapped with ORF0 and ORF2 encodes a serine protease (P1) and possesses a VPg linked to the 5′ end of the genomic RNA. A putative P1–P2 fusion protein, carrying the viral RNA-dependent RNA-polymerase (RdRp) domains, can be produced by ORF1 and ORF2 through a low frequency (−1) ribosomal frameshift event [11,12,13]. The ORF3 and ORF4 encode viral coat protein (CP) and movement protein (MP), respectively [14]. The ORF5 is expressed as a P3–P5 read-through protein via a translational read-through mechanism, which was implicated in aphid transmission and specific virus targeting to phloem [15]. Recently, a new ORF, termed ORF3a, was found to locate upstream of ORF3 and translated from a non-standard (not AUG) start codon. The protein encoded by ORF3a was reported to be required for long-distance movement of the virus in the plant [16].

Maize yellow dwarf virus-RMV (MYDV-RMV) is a new identified species in the genus *Polerovirus* based on ICTV classification (2014 Release, [17]). It was previously known as a Montana isolate of the RMV strain of Barley Yellow Dwarf Virus (BYDV-RMV) listed among unassigned species in the family *Luteoviridae* [4]. BYDV-RMV is most efficiently vectored by the aphid *Rhopalosiphum maidis* and causes Yellow Dwarf diseases in various cereal crops. MYDV-RMV is the only member of BYDV-RMV, whose complete viral genome had been sequenced. For other BYDV-RMV isolates, only the nucleotide region encoding the CP is available.

The traditional methods for virus diagnostics rely mainly on the virus-specific serological or genome-based approaches, such as ELISA and PCR, respectively. Recently, a novel, unbiased approach for plant virus identification has been developed through deep sequencing of virus-derived small interfering RNAs (siRNAs) followed by *de novo* assembly of the complete viral genomes [18,19,20,21,22,23]. This approach requires no prior knowledge of the host or viral nucleotide sequences because the siRNAs are generated during an antiviral defense response in the plants infected by any virus [24,25,26,27]. The virus-specific siRNAs are overlapping in sequence and, thus, can be assembled into long, contiguous fragments (contigs) of the invading viral genome [23]. Previously, the siRNA sequencing has been used for detecting a variety of RNA and DNA viruses including Cereal Yellow Dwarf Virus (CYDV), a polerovirus infecting wild cocksfoot grass (*Dactylis glomerata*) [28].

Here we describe the identification of a virus associated with leaf yellowing and mild mosaic symptoms in the field-grown maize using a high-throughput sequencing of small RNAs (sRNAs). Furthermore, we determine the entire 5642 nucleotide (nt)-long sequence of the viral genome, which shared the highest nucleotide sequence identity (73%) to MYDV-RMV. The genomic organization and sequence analysis strongly support the identification of a novel viral species provisionally designated as Maize yellow mosaic virus (MaYMV) that should be classified in the genus *Polerovirus* of the family *Luteoviridae* together with the demonstration of silencing suppression activity of P0 protein. In view of the fact that MYDV-RMV is fully sequenced from *Triticum aestivum*, to our knowledge, this is the first report of a complete poleroviral genome directly isolated from field maize.

## 2. Materials and Methods

### 2.1. Samples Collection and RNA Isolation

**Figure 1:**
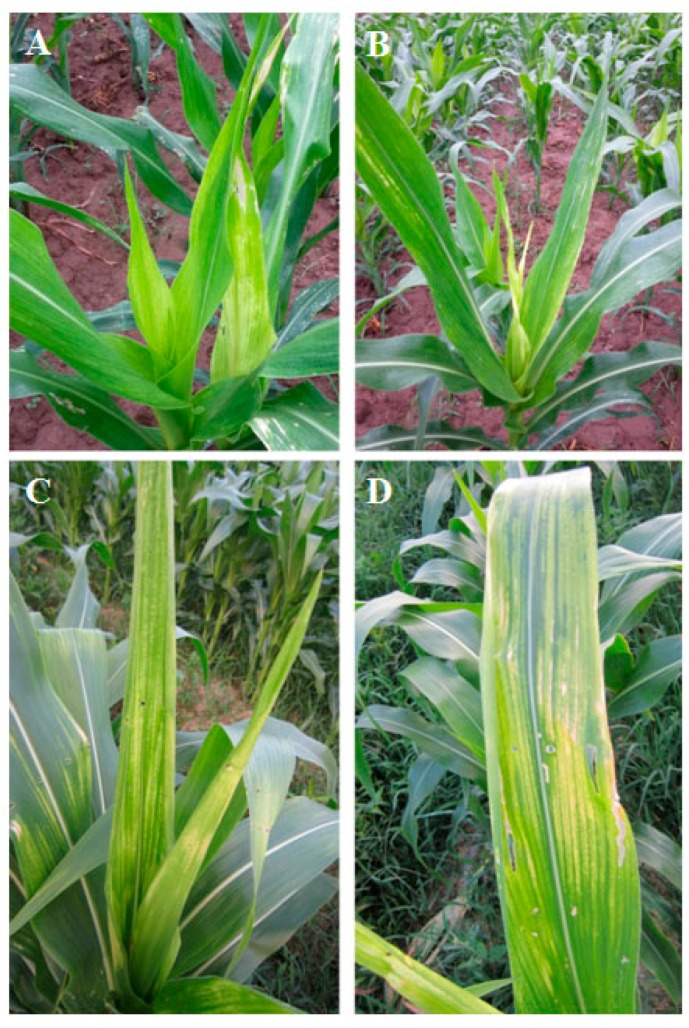
Typical yellow mosaic symptom as represented on the field maizes. Overall view of the whole plants collected from Yuanmou, Yunnan (**A**,**B**) and Changshun, Guizhou (**C**). (**D**) A closer photograph of a leaf associated with MaYMV.

For the field survey of potential viral pathogens of maize (*Zea mays* L.), a total of 49 samples from symptomatic leaves were collected from Yuanmou and Yuanjiang counties in Yunnan Province, as well as Dushan and Changshun counties in Guizhou Province, from January 2013 to May 2014 (Figure 1 and Table 1). Total RNA was extracted using TRIzol reagent (Invitrogen, Carlsbad, CA, USA) from maize leaves following the manufacturer’s guidelines.

### 2.2. Small RNA Library Construction, Sequencing, and Data Processing

The sRNA library was constructed as previously described [29]. Deep sequencing was performed according to the manufacturers’ instructions using the Illumina Hiseq2000 sequencing platform (Illumina, San Diego, CA, USA). Data processing was carried out using a custom bioinformatics pipeline. Raw reads were filtered and cleaned by removing adaptor sequences, poly A reads, and low quality reads with an in-house Perl script. The 18–28 nt clean reads consisting of trimmed sRNA sequences were collected for subsequent analysis. The Velvet program [30] was applied for genome assembly and a parameter of 17 nucleotides was set as the minimal overlapping length (k-mer) required for joining sRNAs into larger contigs [23]. The assembled contigs were subsequently compared against the database of non-redundant (nr) nucleotide sequences from the National Center for Biotechnology Information (NCBI) using a BLASTn (nucleotide blast) search by standard parameters [23].

### 2.3. RT-PCR Validation, Full-Length Genome Amplification, and Sequencing

**Figure 2:**
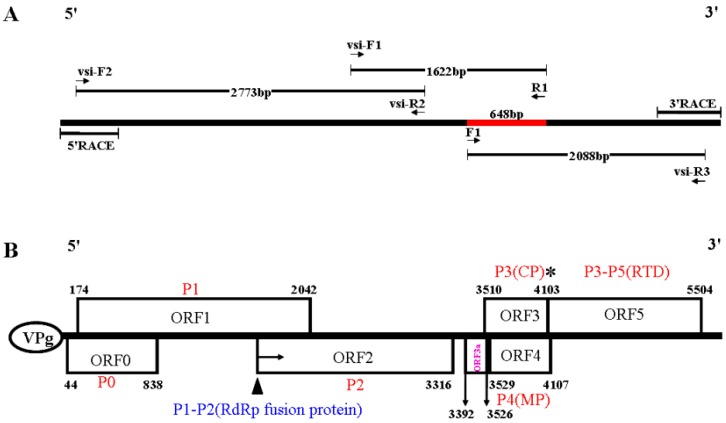
(**A**) Schematic diagram of MaYMV cDNA cloning strategy. The relative positions of primers and RT-PCR products are shown as arrows and line segments, respectively. Vsi designation corresponds to primers that were designed based on virus-derived siRNA sequences. The red line represents the size and position for initially validation of MaYMV by using primer pairs F1/R1; and (**B**) a schematic representation of the genome organization of MaYMV. The predicted frameshift heptanucleotide is marked with a filled triangle, and the “leaky” amber stop codon with an asterisk. RdRp, RNA-dependent RNA polymerase; CP, coat protein; MP, movement protein; RTD, readthrough domain.

To verify the presence of viruses in field samples, RT-PCR was performed as described [31] using primers (Table S1) designed based on the sequence of specific assembled contigs of virus-derived small interfering RNAs (vsiRNAs) for amplification of regions of the MaYMV genome (Figure 2A). The 5′- and 3′-terminal sequences of the MaYMV were obtained through a RACE using SMARTer^™^ RACE cDNA Amplification Kit (Clontech, Mountain View, CA, USA) according to the manufacturer’s protocol. The resulting PCR products were cloned using a ZeroBack Fast Ligation Kit (Tiangen Biotech, Beijing, China) and sequenced via conventional Sanger dideoxy sequencing (Perkin Elmer, Wellesley, MA, USA). The DNAStar Lasergene package (Version 7.1.0, DNAStar Inc., Madison, WI, USA) was used for the full-length genome assembly of the MaYMV.

### 2.4. Sequence and Molecular Variation Analyses

Open reading frames (ORFs) were predicted using the ORF finder function of the Snap Gene software [32]. Multiple alignments of nucleotide sequences were conducted using Clustal W [33] using the neighbor-joining method [34]. Alignment of amino acid sequences was performed using the MUSCLE algorithm [35]. Other parameters were set to the default value. MegAlign software (DNAStar Lasergene package, Version 7.1.0) was performed to analyze the percent nucleotide and deduced amino acid sequence identities between ORFs of MaYMV and other members of the *Luteoviridae*.

The extent of MaYMV variation among these sequences was evaluated using the index π [36] by DnaSP version 5.0 [37], with a sliding window of 100 nt and a step size of 25 nt. The parameter Pi (π) is the mean number of nucleotide differences per site between two sequences to measure the nucleotide diversity [36]. MegAlign software was used to estimate the sequence similarities among the 13 different MaYMV isolates and calculate the ratio of pairwise nonsynonymous (dn) to synonymous (ds) nucleotide substitutions per site.

The phylogenetic trees derived from multiple alignments of the various sequences were conducted using the neighbor-joining method by MEGA6 [38], with the bootstrap of 1000 replicates [39]. Parsimony and maximum likelihood resulted in similar phylogenetic trees. Selected sequences used in the phylogenetic analysis are listed in Table S2.

### 2.5. Assay of PTGS Suppression Function of P0 Protein Encoded by MaYMV

Analysis of the RNA-silencing suppressor was carried out mainly as previously described [31]. Viral cDNAs from the MaYMV-positive sample were used as templates for the RT-PCR amplification of the P0 gene. The resulting fragment, digested with SalI and KpnI, was inserted into pCHF3 to produce 35S-P0. The construct was electroporated into *A. tumefaciens* strain C58C1 and used for the co-infiltration assays. 35S-P19 or 35S-GFP, capable of constitutively expressing Tomato Bushy Stunt Virus p19 and GFP under the control of the Cauliflower Mosaic Virus (CaMV) 35S promoter, were constructed previously [31]. Transgenic *N. benthamiana* plants expressing green fluorescent protein (GFP, line 16c) were infiltrated with a mixture of *Agrobacterium* harboring 35S-GFP and either a test or a control construct. The construct 35S-P19 or empty vector (pCHF3) containing no insert were used as positive and negative controls, respectively.

Silencing of the GFP expression in inoculated and systemic young leaves were monitored through continuous observation under a UV lamp (UV Products, Upland, CA, USA) after three days post inoculation (dpi). Tissue samples (100 mg) were collected for immunoblot analysis at 5 and 20 dpi, respectively. Accumulation of GFP in infiltrated and systemic leaves was evaluated using a GFP-specific antibody as previously described [31]. Total RNA was extracted using TRIzol reagent (Invitrogen), and Northern blot analysis was conducted using the DIG High Prime DNA Labeling and Detection Starter Kit II, according to the manufacturer’s instructions (Roche Diagnostics GmbH, Mannheim, Germany). Membranes were hybridized separately to DIG-labeled probes of the GFP gene.

### 2.6. Construction of cDNA Clones of MaYMV, Agro-Infiltration, and Detection of Viral RNA

The complete nucleotide sequence of MaYMV was produced using high-fidelity DNA polymerase (New England Biolabs, Ipswich, MA, USA) with specific primer pair Full-F/Full-R (Table S1). The resulting PCR fragment was inserted into a modified pCB301 vector with 2 × 35S promoter from CaMV (kindly provided by Xiaorong Tao) using the ClonExpress^®^ II One Step Cloning Kit (Vazyme, Nanjing, China) resulting in the pCB301-MaYMV plasmid. The plasmid DNA was introduced into the *A. tumefaciens* strain C58C1 by electro-transformation [40].

The recombinant *A. tumefaciens* cells were incubated in inoculation buffer (10 mM MES, 10 mM MgSO4, and 100 mM acetosyringone). Subsequently, the bacterial culture was infiltrated into the abaxial surface of the two-week-old *N. benthamiana* leaves [41], whereas an empty pCB301 vector was used as a negative control.

Total RNAs were isolated from upper non-inoculated leaves three, six, and nine dpi and were subsequently subjected to RT-PCR and Northern blot analysis for virus detection. Northern blot analysis was carried out following the procedures described previously [42]. Viral-specific primers F2/R2 used for RT-PCR and amplification for probe used in Northern blot assays are listed in Table S1.

## 3. Results

### 3.1. Analysis of sRNA Library and Identification of Virus in Maize by *de Novo* Assembly of vsiRNAs

A sRNA library, from a maize sample with yellowing and mild mosaic symptoms collected from Yuanmou county, Yunnan province of China (Figure 1), was constructed and sequenced using the Illumina Hiseq2000 platform. As a result, 10,781,056 clean reads were produced after removing adaptor sequences and filtering for poly A reads and low quality reads. The nucleotide sequences of 10,739,457 sRNA of 18–28 nt in length were obtained using an in-house Perl script (Table S3) and used to assemble contigs [30], 536 of which have been identified. BLASTn analysis of the assembled contigs against sequences in the NCBI database, using a highly homology sequence search, identified 11 contigs mapping to several Yellow Dwarf Viruses (YDVs) of the family *Luteoviridae* (Table S4). Importantly, five of these 11 contigs were most similar to MYDV-RMV (GenBank accession number NC_021484) with a nucleotide sequence identity of 86%–93%, whereas the remaining six contigs matched closely the CP-encoding sequence from other BYDV-RMV isolates with a nucleotide sequence identity of 95%–99% (Table S4).

### 3.2. The Complete Nucleotide Sequence and Genome Organization of a Novel RNA Virus

The presence of virus in field maize sample was further validated by sequencing a 648 bp fragment amplified with a forward primer F1 (identical to the 5′-terminal sequence out of BYDV-RMV-Illinois CP) and a reverse primer R1 (complementary to the selected contigs NODE_252, see Table S4). This sequence was most closely related to the CP sequence of BYDV-RMV-Illinois or BYDV-RMV-Brazil isolates (GenBank accession number: Z14123 and JX067855) with a nucleotide sequence identity of 94.6% to 98%; however, for the latter, only a partial CP sequence is available. Subsequently, RT-PCR was performed to obtain the full-length genomic sequence of this virus using primers designed based on the sequence of the vsiRNAs (Table S1). A schematic of the cloning strategy used for isolating the full-length genome is shown in Figure 2A. Three fragments of 2773, 1622 and 2088 bp were obtained using primer combinations of vsi-F2/vsi-R2, vsi-F1/R1, and F1/vsi-R3, respectively. The 5′ and 3′ non-coding regions were determined by 5′ and 3′ RACE-PCRs. After assembling by SeqMan (Lasergene package, Version 7.1.0), the complete genome of this virus was determined to be 5642 nt in length (GenBank accession Number: KU248489). The BLASTn search of the full-length nucleotide sequence against the NCBI database indicated that it had significant similarity to viruses belonging to the genus *Polerovirus* of the family *Luteoviridae*, sharing the closest relationship to MYDV-RMV with 73% identity. These results suggested that a putative novel virus was present in the sequenced sample, and the name Maize Yellow Mosaic Virus (MaYMV) was provisionally introduced.

The ORF Finder predicted six ORFs, namely ORF0, ORF1, ORF2, ORF3, ORF4, ORF5, and three UTRs in the MaYMV genomic sequence (Figure 2B), suggesting the genome organization of MaYMV was striking similar to poleroviruses [6,43]. Meanwhile, the ORF3a, positioned upstream of ORF3, and its translation initiates at an AUA codon, can be predicted at position nucleotides 3392–3526 as described [14], which would give rise to a 45 amino acid P3a product (Figure 2B). The 5′ UTR consists of 44 nt beginning with the sequence ACAAAA, a feature found in most poleroviruses [3,4,5,6]. The 3′ UTR of 177 nt and an intergenic UTR region of 194 nt between ORF2 and ORF3 were predicted, respectively.

The products of seven predicted ORFs in the MaYMV genome had a high amino acid identity with poleroviruses based on pair-wise comparisons with 26 identified members of the *Luteoviridae*, sharing a closest relationship with MYDV-RMV (Table 2). It is worth noting that the RdRp fusion protein produced by ORF1 and ORF2 and CP protein encoded by ORF3 shared the highest identity with those of MYDV-RMV (78.8% and 77.7%).

### 3.3. Phylogenetic Analysis of the MaYMV Genome

**Figure 3:**
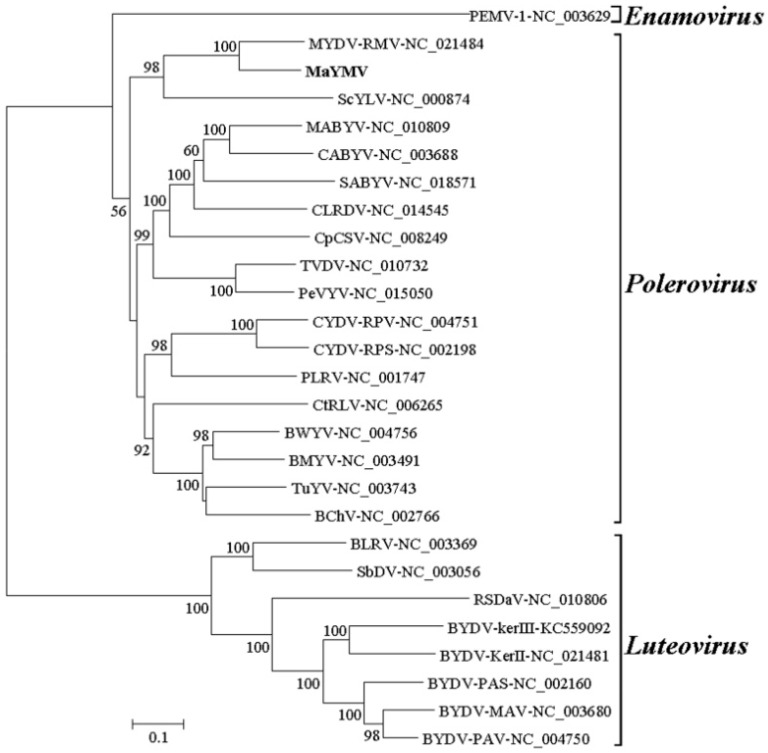
Phylogenetic analyses of MaYMV and selected luteoviruses based on complete genome sequences. The phylogenetic trees were generated using the neighbor-joining method and MEGA6 software. The percentage of replicate trees in which the associated taxa clustered together in the bootstrap test (1000 replicates) is shown next to the branches.

Neighbor-joining phylogenetic analysis based on full-length viral genomes was performed with a bootstrap of 1000 replications. All of the 27 selected sequences were well separated into three clades corresponding to genera *Luteovirus*, *Polerovirus*, and *Enamovirus*. MaYMV grouped confidently with the members in the genus *Polerovirus*, and was most closely related to MYDV-RMV with 73% identity (Figure 3, Table 2). Furthermore, the phylogenetic tree constructed based on the amino acid sequences of either RdRp or CP, which are widely used as important criteria for assigning virus species to a genus in the family *Luteoviridae*, supported MaYMV grouping with the poleroviruses within a small cluster containing BYDV-RMV isolates (Figure S1, Table 2). Taken together, these results clearly suggest that MaYMV might be a new member of the genus *Polerovirus*.

### 3.4. Distribution of vsiRNAs Along the MCMV Genome

**Figure 4:**
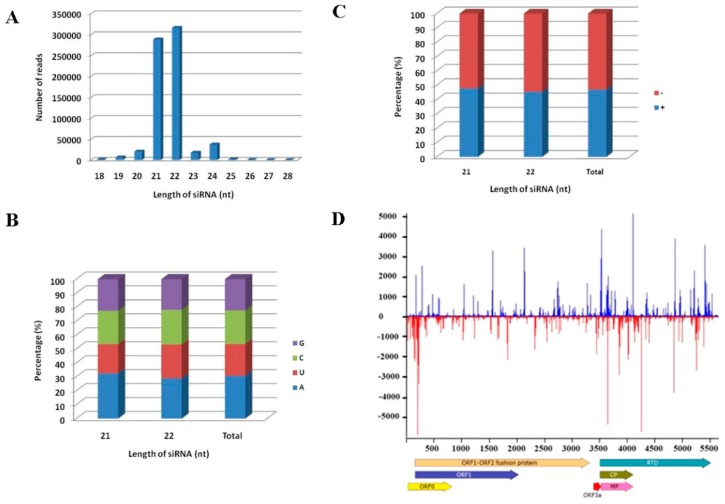
Characterization of the MaYMV vsiRNAs. (**A**) Size distribution of the vsiRNAs along MaYMV genome; (**B**) the relative frequency of the four different 5′ terminal nucleotides in vsiRNAs; (**C**) polarity distribution of vsiRNAs that perfectly matched either plus or minus MaYMV genomic sequence; and (**D**) distribution profile of the vsiRNAs that revealed multiple hotspots along the MaYMV genome.

To get an insight into the host RNA silencing defense induced by MaYMV, the profile of siRNAs derived from MaYMV (MaYMV-vsiRNAs) was characterized using bowtie tools and allowed zero mismatches [44]. A total of 687,603 reads were perfectly mapped along MaYMV genome, which account for 6.4% of the total sRNAs (Table S3). These MaYMV-vsiRNAs were mostly dominated by 21- and 22-nt species (Figure 4A), accounting respectively for 41.89% and 45.96% of the total reads. In addition, an adenosine (A) residue was preferentially used at the 5′ terminal position in 21- and 22-nt MaYMV-vsiRNAs, corresponding to 32.49% and 28.84% A of the first nucleotides, respectively (Figure 4B). When the total MaYMV-vsiRNAs were mapped to the MaYMV genome, a slight asymmetric distribution resulting in a moderate excess (about 53%) towards the complementary strand was observed (Figure 4C). The MaYMV-vsiRNAs covers nearly the entire genome of MaYMV (Figure 4D) with a distribution profile exhibiting numerous hot spots in both genome orientations. These hot spots were particularly prominent in the 5′-terminal and the MP/CP/RTD-coding regions (Figure 4D).

### 3.5. The P0 Protein Encoded by MaYMV Inhibits Local and Systemic RNA Silencing

**Figure 5:**
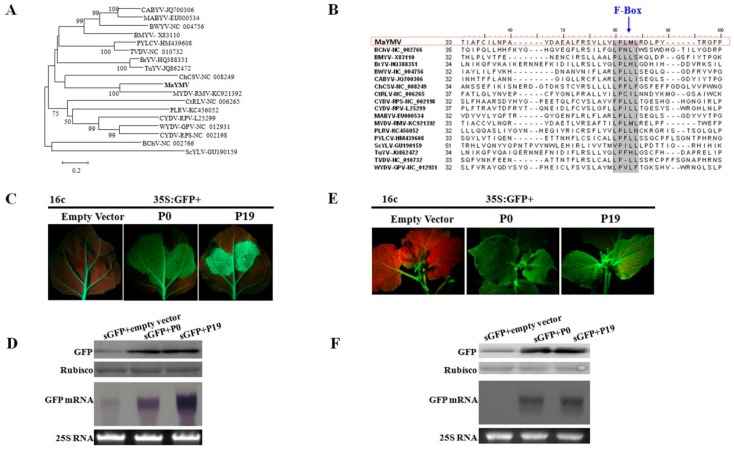
Analysis of the local and systemic silencing of GFP mRNA in transgenic *N. benthamiana* line 16c using ectopic expression of the MaYMV P0 in an agro-coinfiltration assay. (**A**) amino acid sequences of the P0s encoded by polereoviruses were aligned using the MUSCLE algorithm. The aligned sequences were used for inferring evolutionary relationships using the neighbor-joining method. The resulting tree, drawn to scale, is shown with the bootstrap of 1000 replicates; (**B**) multiple alignment of the polerovirus P0 amino acid sequences encompassing a conserved F-box motif LPxxL/I; (**C**) suppression of local silencing by MaYMV P0 protein. Images of infiltrated leaves were taken under long wave length UV light at 5 dpi; (**D**) Western blot analyses of GFP accumulation (in top panels) or Northern blot analyses of GFP mRNA levels (in bottom panels) in the extracts from the agro-infiltrated leaves are shown in (**C**); (**E**) suppression of systemic silencing by MaYMV P0 protein. The agro-infiltrated plants were imaged at 20 dpi. The infiltration combinations of sGFP with either empty vector or P19 were used as negative and positive cocontrols, respectively; and (**F**) immunoblot analyses of GFP accumulation (in top panels) or Northern blot analyses of GFP mRNA levels (in bottom panels) in the extracts from the systemic leaves are shown in (**E**).

The P0 protein encoded by the 5′-terminal ORF in poleroviruses generally shows a high level of amino acid sequence variability among the members of this genus (Table 2). In particular, MaYMV P0 is most closely related to MYDV-RMV P0 with only 48% of sequence identity (Table 2). A closer examination of P0 amino acid sequences revealed conservation of the F-Box-like motif among all poleroviruses including MaYMV (Figure 5A,B) and suggesting its putative RSSs function. The local and systemic silencing suppression activity of MaYMV P0 was investigated in agro-coinfiltration assays using the 16c transgenic *N. benthamiana* plants. At 5 dpi, strong green fluorescence was observed in leaves co-infiltrated with 35S-GFP plus either 35S-P0 or 35S-P19. In contrast, the infiltrated areas of negative control leaves appeared as a red patch suggesting complete GFP silencing (Figure 5C). Immunoblot analysis with a GFP-specific antibody was used to validate correlation between the observed GFP fluorescence and GFP accumulation (Figure 5D). As expected, GFP was readily detected in the 35S-P0 or 35S-P19 infiltrated plants, with higher levels in the leaves tested at 5 dpi than in the leaves co-infiltrated with 35S-GFP plus empty vector. Similarly, GFP mRNA accumulated to higher levels in leaves co-infiltrated with 35S-GFP plus either 35S-P0 or 35S-P19, whereas GFP mRNA levels were remarkably reduced in negative control leaves (Figure 5D). These results indicated that the MaYMV P0 possesses RSS activity that acts at a local level.

To explore the effect of MaYMV P0 on systemic spread of RNA silencing, the infiltrated 16c plants were monitored for GFP expression in the newly emerging upper leaves that were not agroinfiltrated. At 20 dpi, 19 of 23 plants agro-coinfiltrated with 35S-GFP plus the empty vector showed obvious silencing in systemic leaves, whereas P0 inhibited the systemic spread of the silencing in 20 of 23 plants producing GFP fluorescence similar to that induced by 35S-GFP plus 35S-P19 (Figure 5E). These findings were further confirmed by Western blot and Northern blot analyses (Figure 5F). Taken together, our results demonstrate that the MaYMV P0 is a strong suppressor of both local and long-distance RNA silencing.

### 3.6. Infectivity Assays in N. Benthamiana Plants

**Figure 6:**
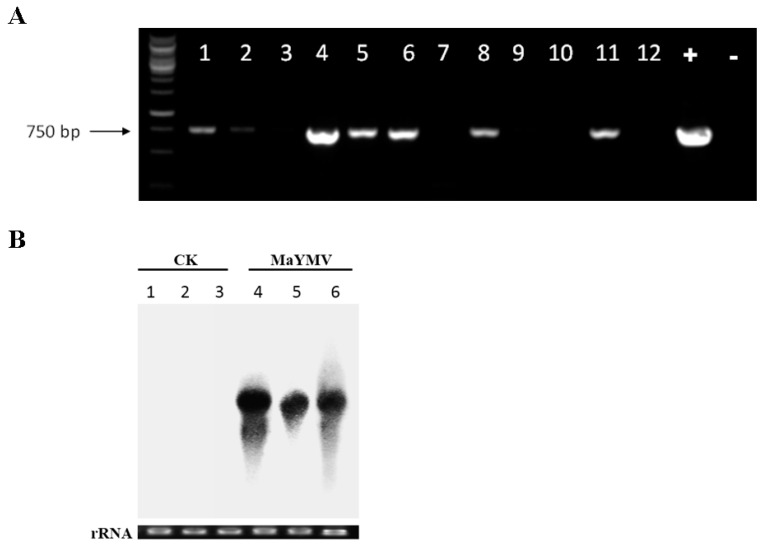
Agroinoculation infectivity assays using pCB301-MaYMV and *N. benthamiana* plants. (**A**) RT-PCR analysis of not inoculated (systemic) leaves infected by MaYMV. The numbers from 1 to 12 correspond to individual plants inoculated with pCB301-MaYMV infectious clone. “+” represents the positive control (using plasmid pCB301-MaYMV as a PCR template); “−“ represents the negative control (using the cDNA from mock-inoculated *N. benthamiana* as a PCR template); and (**B**) Northern blot analysis of MaYMV RNA obtained from pCB301-MaYMV-agroinoculated upper leaves of plants and the MaYMV CP ORF-derived cDNA probes. Equal loading of total RNA (20 μg) was validated by ethidium bromide-stained agarose gels as indicated at the bottom of the panel. Numbers 1–3 correspond to the replicates of the negative control; numbers 4–6 are the replicates of samples from plants agro-inoculated with pCB301-MaYMV.

The full-length cDNA clone of MaYMV, pCB301-MaYMV, was constructed and used for infectivity tests done by agroinoculation of the *N. benthamiana* plants. The RT-PCR and Northern blot analyses showed accumulation of the viral RNA in the non-inoculated (systemic) leaves at 6 dpi, but not in the mock-inoculated control (CK) plants (Figure 6A,B). These results validated the biological activity of the pCB301-MaYMV cDNA clone that elicited full-scale systemic infection in an experimental host, *N. benthamiana*. Interestingly, no detectable disease symptoms were present in this host.

The percentage of infection caused by pCB301-MaYMV was further analyzed by RT-PCR. Three independent experiments detected MaYMV RNA in systemically-infected leaves at three dpi, with an average infection efficiency rate of about 7%. At nine dpi, infection efficiency has increased to nearly 90%.

### 3.7. Field Detection of MaYMV

A total of 49 maize leaf samples from fields showing yellowing and mosaic symptoms from Yunnan and Guizhou regions were investigated for the presence of MaYMV using RT-PCR assay with primer pair F1/R1 (the positive band was further identified by sequencing, Table S1). The 37 of the samples were positive for MaYMV (Table 1). Further the 37 MaYMV-positive samples were tested for the presence of Maize Chlorotic Mottle Virus (MCMV), Sugarcane Mosaic Virus (SCMV), and Southern Rice Black-Streaked Dwarf Virus (SRBSDV), which are widespread on maize plants in Southern China. This analysis demonstrated that at least one additional virus was present in many samples indicating wide spread of mixed infections involving MaYMV (Table 1).

### 3.8. Molecular Variation of the MaYMV Genome

**Figure 7:**
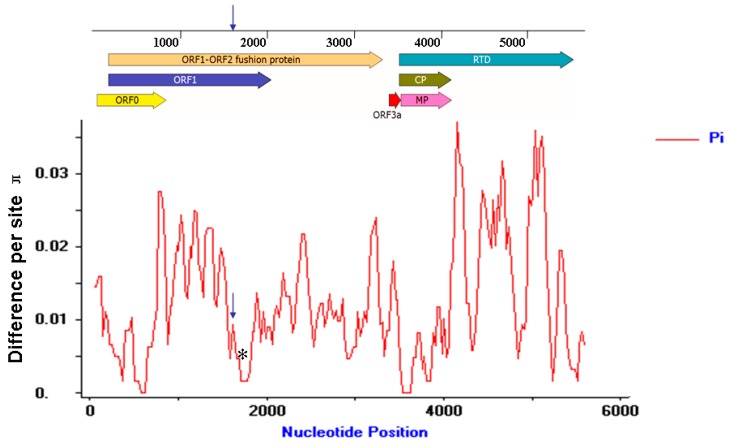
Distribution of MaYMV genetic variation estimated by nucleotide diversity (π). The sliding window was 100 sites wide with slide set at 25-site intervals. The relative positions of the seven ORFs of the MaYMV genome are illustrated by lines above the plot. The positions of conserved frameshifting sequence and pseudoknot sequences are shown as blue arrow and black asterisks, respectively.

Of the 37 MaYMV-infected maize leaves samples described above, 12 samples were further randomly selected and used for whole genome cloning and sequencing. Pair-wise nucleotide sequence alignments performed by MegAlign software demonstrated an identity from 98.3% to 99.5% among the total 13 different MaYMV isolates (GenBank Accession: KU179221, KU248490, KU248489, KU291099-KU291108). To further evaluate the extent of MaYMV genome variation, the average pairwise diversity parameter π was introduced to calculate at all sites along the MaYMV genome by DnaSP Version 5.0 software (Figure 7). These results showed that the overall nucleotide variability for the MaYMV genome was below 4%, and an obvious fluctuation of genomic diversity was observed. The peak of variability was present within the 3′ half of the P3–P5 read-through protein-coding region, whereas the lowest level of nucleotide diversity was observed in the CP and MP-coding genome regions (Figure 7).

Further insight into MaYMV variability was obtained by measuring the specific mutational changes using the residue substitution functions of MegAlign. The residue substitutions of T-C and A-G were overwhelmingly represented, comprising together 91.2% of the mutations. The other mutational changes, such as transversions of A–T, A–C, T–G, and C–G, were underrepresented, collectively comprising only 8.8% of all mutations (Table S5). These results indicated that transition biases appear to exist during the short-term evolution of MaYMV.

## 4. Discussion

All members of the *Luteoviridae* are of significant economic importance [45,46]. The family *Luteoviridae* currently includes 26 virus species in three genera. Here, a novel polerovirus belonging to the family *Luteoviridae* was identified in a crop plant of paramount economic value, the maize. This has been done using an advanced deep sequencing of vsiRNAs aided by direct sequencing of RT-PCR products generated to close the gaps between contigs derived from vsiRNA sequence assembly. Finally, the 5′- and 3′-terminal sequences of viral genome were obtained using 5′ and 3′ RACE-PCR, and a full-length genome of MaYMV was assembled as a combined result of these three complementary approaches.

The following analysis of the MaYMV genome organization showed typical features and key cis-acting elements conserved in other poleroviruses. The 5′ UTR of MaYMV begins with ACAAAA, a signal for RNA recognition by RdRp that is common in poleroviruses [4,5,6,47,48]. Similarly, MaYMV contains a copy of the ACAAAA consensus sequence upstream of the start of the intergenic region previously identified as a transcription start site for the subgenomic RNA1 (sgRNA1) of Chickpea Chlorotic Stunt Virus (CpCSV), Beet Western Yellow Virus (BWYV), and MYDV-RMV [4,43]. The same transcriptional signal found 202 nt upstream from the start of the coat protein ORF encoded by MaYMV, similar to that of MYDV-RMV [4]. Furthermore, a (CCXXXX)16 repeat motif present at MaYMV nucleotides 4119–4214 is reminiscent of (CCXXXX)12 motif at nucleotides 4131–4199 of MYDV-RMV, where it was implicated in read-through of the CP ORF stop codon [4]. Thus, the MaYMV genome likely shares the same −1 ribosomal frameshift and read-through translation strategies with other members of the genus *Polerovirus*.

The full-length nucleotide sequence comparisons showed that MaYMV shares the highest level of sequence identity of 73% with a Montana isolate of MYDV-RMV, collected from *Triticum aestivum*, whereas the amino acid sequence of any gene products differs by more than 10% between them. The phylogenetic analyses on the basis of both complete nucleotide sequences and amino acid sequences encoding for CP and RdRp demonstrated that MaYMV groups with the poleroviruses, finalizing its taxonomic classification with this genus. Thus, according to the criteria for species demarcation in the family *Luteoviridae* by the ninth report of the ICTV [2], we propose MaYMV to be considered as a new species in the genus *Polerovirus* of the family *Luterviridae*. However, it is noticed that the phylogenetic tree based on the CP amino acid sequence also shows that MaYMV have the closest relation with BYDV-RMV Illinois and Brazil isolates (identity ranged from 94.7%–98.3%), for which only the CP gene sequences are available. Hence, MaYMV might be most similar to BYDV-RMV Illinois and Brazil isolates in genome parts other than the CP gene.

Our analysis of MaYMV-induced vsiRNAs showed that most of them belonged to 21- and 22-nt classes, suggesting that the maize orthologs of Dicer-like (DCL) 4 and 2 likely mediate their genesis, similar to the previous reports in distinct virus-plant models [21,49,50,51]. We have also found a bias toward the identity of the first 5′-nucleotide of the MaYMV vsiRNAs, in accord with the role played by this nucleotide in recruiting of siRNAs into specific AGO complexes [52,53]. This bias for the enrichment in adenosine at the 5′ end of vsiRNAs is statistically significant when compared to the adenosine content in the MaYMV genome [54], suggesting that MaYMV vsiRNAs are prevalently recruited by the AGO2 and AGO4 complexes.

The PTGS in plants is a major and universally-conserved mechanism of the antiviral defense [55,56,57]. In a counter defensive response, many plant viruses acquired diverse RSSs. In particular, several P0 proteins of poleroviruses have been shown to possess a strong RSSs activity [7,8,9]. Despite a remarkable overall variability of the P0 amino acid sequences, a short conserved F-box-like motif (LPxxL/I) has been implicated in P0 suppressor activity [58]. As we demonstrated in this work, the MaYMV P0 does also harbor an F-box-like motif and displays RSS activity in both local and systemic silencing. However, unlike some of the polerovirus P0s that elicit a hypersensitive response or severe systemic necrosis in *Nicotiana* species [9,59], the ectopically-expressed MaYMV P0 did not exert such responses in *Nicotiana benthamiana*.

To enable future reverse genetic studied of the MaYMV functional genomics, we have developed a biologically-active cDNA clone. Furthermore, to overcome the lack of mechanical transmissibility of poleroviruses that prevents plant infection via RNA transcript inoculation, we adopted agroinfection whereby the virus replication is launched from the T-DNA delivered to nuclei by agrobacterium [60]. This approach using a binary vector pCB301-MaYMV was successfully applied for systemic, albeit symptomless infection of an experimental host *N. benthamiana*. Similar results were previously reported for other poleroviruses including BYDV-PAV, Cereal Yellow Dwarf Virus-RPV (CYDV-RPV) and Brassica Yellows Virus [61,62]. However, other *Nicotiana* species, such as *N. tabacum* and *N. glutinosa* could not be infected by agroinoculation of pCB301-MaYMV, in agreement with earlier work on Beet Mild Yellowing Virus [63]. Our attempts to agroinoculate maize and oat plants were also unsuccessful, consistent with the previous studies of BYDV-PAV and CYDV-RPV [61] and general recalcitrance of the monocot plants to agroinoculation.

## 5. Conclusions

In this work we describe the discovery of MaYMV, a novel maize polerovirus that is broadly distributed in Southern China, often in mixed infections with other RNA viruses. We further characterize the MaYMV genome, its natural variability, as well as vsiRNAs induced in maize plants in response to virus infection. Finally, we generate a biologically-active MaYMV cDNA clone capable of systemic infection of *N. benthamiana*. An additional effort is needed, however, to develop a suitable technology for applying this clone for MaYMV studies in its natural host, maize.

## Figures and Tables

**Table 1:** Detection of mixed infections in maize samples collected from Yunnan region of China.

Location	Year	Sample No.	GenBank No.	Virus			
				MaYMV	MCMV	SCMV	SRBSDV
Yuanjiang, Yunnan	2013	YJ-1	/	+	+	+	−
		YJ-3	KU179221	+	−	+	+
		YJ-4	KU248490	+	−	+	+
		YJ-5	KU291099	+	+	+	−
		YJ-6	KU291100	+	+	+	−
		YJ-7	KU291101	+	−	+	−
Yuanmou, Yunnan	2014	YM-1	KU291102	+	+	−	−
		YM-2	KU291104	+	+	+	−
		YM-3	/	+	+	−	−
		YM-4	/	+	+	+	−
		YM-5	/	+	+	+	−
		YM-6	KU291106	+	+	−	−
		YM-7	/	+	+	+	−
		YM-8	/	+	−	−	−
		YM-9	/	+	−	−	−
		YM-11	KU291103	+	+	−	−
		YM-12	/	+	+	−	−
		YM-14	/	+	+	−	−
		YM-15	KU248489	+	−	−	−
		YM-16	/	+	−	−	−
		YM-18	/	+	−	−	−
		YM-19	/	+	+	−	−
		YM-20	KU291105	+	+	−	−
		YM-21	/	+	+	−	−
		YM-22	/	+	+	−	−
Changshun, Guizhou	2014	Csh-1	KU291107	+	−	−	−
		Csh-2	/	+	−	−	−
		Csh-3	KU291108	+	−	−	−
		Csh-5	/	+	−	−	−
		Csh-7	/	+	−	−	−
		Csh-8	/	+	−	−	−
		Csh-9	/	+	−	−	−
		Csh-11	/	+	−	−	−
		Csh-12	/	+	−	−	−
		Csh-14	/	+	−	−	−
Dushan, Guizhou	2014	Dsh-4	/	+	−	−	−
		Dsh-5	/	+	−	−	−

“−” indicates a negative result of viral detection.

**Table 2:** Percent nucleotide and amino acid sequence identity between ORFs of MaYMV and other members of the *Luteoviridae*.

Genus	Virus Abbreviation-GenBank Number	Genome Size(nt)	Nucleotide Identity (%)	Amino Acid Similarity (%)						
				P0	P1	RdRp (P1–P2)	CP (P3)	ORF3a (P3a)	MP (P4)	RTD (P3–P5)
*Luteovirus*	BLRV-NC_003369	5964	38.0	NA	6.8	9.3	49.2	34.9	46.5	30.7
*Luteovirus*	SbDV-NC_003056	5853	36.8	NA	7.5	9.9	53.9	44.4	42.7	30.5
*Luteovirus*	RSDaV-NC_010806	5808	33.7	NA	6.0	8.3	36.0	48.9	25.4	28.8
*Luteovirus*	BYDV-KerII-NC_021481	5736	36.2	NA	8.2	8.6	45.0	35.6	28.9	31.5
*Luteovirus*	BYDV-PAS-NC_002160	5695	36.2	NA	11.1	8.1	42.8	35.6	27.5	33.4
*Luteovirus*	BYDV-kerIII-KC559092	4625	38.1	NA	10.9	10.1	41.7	37.8	23.0	31.7
*Luteovirus*	BYDV-PAV-NC_004750	5677	37.2	NA	8.5	8.4	43.8	35.6	26.1	33.1
*Luteovirus*	BYDV-MAV-NC_003680	5273	37.4	NA	9.1	8.4	44.6	33.3	25.3	32.4
*Polerovirus*	BMYV-NC_003491	5722	50.3	20.1	33.2	50.8	59.9	65.6	42.8	34.8
*Polerovirus*	BWYV-NC_004756	5666	50.0	20.0	32.6	49.0	60.2	53.3	39.9	32.9
*Polerovirus*	BChV-NC_002766	5776	48.8	16.9	25.7	43.6	61.7	65.6	38.2	33.0
*Polerovirus*	SABYV-NC_018571	5843	49.5	19.8	26.0	41.2	59.2	55.6	42.9	39.3
*Polerovirus*	CLRDV-NC_014545	5866	50.3	18.5	30.0	48.4	62.2	55.6	44.8	38.5
*Polerovirus*	CABYV-NC_003688	5669	51.1	19.7	32.7	48.3	59.5	55.6	43.2	40.5
*Polerovirus*	MABYV-NC_010809	5674	51.6	21.5	33.7	49.4	57.9	53.3	45.5	38.5
*Polerovirus*	CpCSV-NC_008249	5900	53.0	17.4	29.4	45.2	61.7	46.7	37.0	39.9
*Polerovirus*	PeVYV-NC_015050	6244	49.1	21.0	29.7	47.7	54.8	48.9	31.6	36.6
*Polerovirus*	TVDV-NC_010732	5920	47.8	20.9	31.9	49.1	53.8	48.9	31.8	32.7
*Polerovirus*	CtRLV-NC_006265	5723	47.4	13.6	29.6	46.9	44.4	51.1	27.4	29.0
*Polerovirus*	PLRV-NC_001747	5987	49.6	15.2	27.6	45.4	57.7	53.3	44.9	35.1
*Polerovirus*	TuYV-NC_003743	5641	51.3	20.1	33.2	49.9	60.9	44.4	42.2	34.9
*Polerovirus*	CYDV-RPS-NC_002198	5662	48.5	15.9	27.8	42.9	58.7	42.2	43.5	34.1
*Polerovirus*	CYDV-RPV-NC_004751	5723	49.1	16.9	28.6	43.0	60.7	40.0	40.1	36.4
*Polerovirus*	ScYLV-NC_000874	5899	56.3	10.2	29.7	44.4	41.0	56.8	32.0	16.7
*Polerovirus*	MYDV-RMV-NC_021484	5612	73.0	48.3	68.3	78.8	77.7	33.3	62.5	60.8
*Enamovirus*	PEMV1-NC_003629	5706	40.3	10.6	16.7	27.8	32.4	NA	NA	42.7

“NA” indicates not applicable.

## Supplementary Materials

The following are available online at www.mdpi.com/1999-4915/8/5/120/s1, Figure S1: Phylogenetic analyses of MaYMV and selected luteoviruses based on amino acid sequences of RdRp or CP, Table S1:primers used in the article, Table S2:Sequences of selected members of the Luteoviridae used in the sequence analysis, Table S3: Deep sequencing data and assembly of small RNAs, Table S4: Contigs found to have identity to Yellow dwarf virusesin a BLASTn search of the NCBI database, Table S5:Percentages of nucleotide substitutions in MaYMV genome.
