# Supplementary material for: Characterization of a Novel Polerovirus Infecting Maize in China
Source: Viruses. 2016 Apr 28;8(5):120. doi: 10.3390/v8050120 (PMC4885075; doi:10.3390/v8050120)
Supplement: Supplementary file 1 [file viruses-08-00120-s001.pdf]

Supplementary Materials: Characterization of a Novel Polerovirus Infecting Maize in China

Sha Chen, Guangzhuang Jiang, Jianxiang Wu, Yong Liu, Yajuan Qian and Xueping Zhou

Table S1. Primers used in the article.

Primer	Sequence(5′→3′)
used for clone MaYMV	
F1	GTACAAGATCTACCTATCTATCTCCTCG
R1	CGTCTACCTATCTCGGATTTTGAACA
F2	TGCTCACATGTCTTCGAGAAGGAA
R2	TCGGAGATGAACTCCAGTATGATCT
vsi-F1	AGGCCGCTACCGCCTCATCAT
vsi-F2	CGATACGATGCAGAAGCTCT
vsi-R2	TCGAACTGGTGTTGTAGGAGCC
vsi-R3	AGGTTGTCATCCTGTCTCGTT
Full-F	TGACCTGCAGGTCGACACAAAAGGCACCCAAGGGA
Full-R	AGATGCCATGCCGACCCGGGACCAAGGATTTCAGGGGA
used for mix-infection detection in field samples	
MaYMV-F	TGCTCACATGTCTTCGAGAAGGAA
MaYMV-R	TCGGAGATGAACTCCAGTATGATCT
MCMV-F	TCACCGATCCAGTAGCCATTGGAT
MCMV-R	TCAAGATGAACAGAATCGAGGAGATCA
SCMV-F	AGTGGCGCAACAAAGTGTGGATGT
SCMV-R	TCTCGGCTAGTGGTCTTGTTGGT
SRBSDV-F	TGTCTGCACTTAGAAAAGGAGTCG
SRBSDV-R	TAGCTTTCTTAATTAGACGATCGCGA

Table S2. Sequences of selected members of the *Luteoviridae* used in the sequence analysis.

Classification	Virus Name	Abbreviation	Accession No.
Luteovirus	Bean leafroll virus	BLRV	NC_003369
Luteovirus	Barley yellow dwarf virus-kerII	BYDV-kerII	NC_021481
Luteovirus	Barley yellow dwarf virus-kerIII	BYDV-kerIII	KC559092
Luteovirus	Barley yellow dwarf virus-MAV	BYDV-MAV	NC_003680
Luteovirus	Barley yellow dwarf virus-PAS	BYDV-PAS	NC_002160
Luteovirus	Barley yellow dwarf virus-PAV	BYDV-PAV	NC_004750
Luteovirus	Rose spring dwarf-associated virus	RSDaV	NC_010806
Luteovirus	Soybean dwarf virus	SbDV	NC_003056
Polerovirus	Beet chlorosis virus	BChV	NC_002766
Polerovirus	Beet mild yellowing virus	BMYV	NC_003491
Polerovirus	Beet western yellows virus	BWYV	NC_004756
Polerovirus	Carrot red leaf virus	CtRLV	NC_006265
Polerovirus	Cereal yellow dwarf virus-RPS	CYDV-RPS	NC_002198
Polerovirus	Cereal yellow dwarf virus-RPV	CYDV-RPV	NC_004751
Polerovirus	Chickpea chlorotic stunt virus	CpCSV	NC_008249
Polerovirus	Cotton leafroll dwarf virus	CLRDV	NC_014545
Polerovirus	Cucurbit aphid-borne yellows virus	CABYV	NC_003688
Polerovirus	Maize yellow dwarf virus-RMV	MYDV-RMV	NC_021484
Polerovirus	Melon aphid-borne yellows virus	MABYV	NC_010809
Polerovirus	Pepper vein yellows virus	PeVYV	NC_015050
Polerovirus	Potato leafroll virus	PLRV	NC_001747

Table S2. *Cont.*

Classification	Virus Name	Abbreviation	Accession No.
Polerovirus	Suakwa aphid-borne yellows virus	SABYV	NC_018571
Polerovirus	Sugarcane yellow leaf virus	ScYLV	NC_000874
Polerovirus	Tobacco vein distorting virus	TVDV	NC_010732
Polerovirus	Turnip yellows virus	TuYV	NC_003743
Enamovirus	Pea enation mosaic virus-1	PEMV-1	NC_003629

Table S3. Deep sequencing data and assembly of small RNAs.

Clean reads	10,781,056
Clean bases	240,323,048
18–28 nt reads	10,739,457(99.6%)
Total contigs after assembling small RNA	536
Contigs matching Barley yellow dwarf virus-RMV, BYDV-RMV	5
Contigs matching Maize yellow dwarf virus-RMV, MYDV-RMV	5

Table S4. Contigs found to have identity to Yellow dwarf viruses in a BLASTn search of the NCBI database.

	Contig Name	Length	Identity
Contigs Mapping Maize yellow dwarf virus-RMV	NODE_26	482	409/475 (86%)
	NODE_38	179	133/153 (87%)
	NODE_69	142	124/140 (89%)
	NODE_111	88	77/88 (88%)
	NODE_199	85	77/83 (93%)
Contigs Mapping Barley yellow dwarf virus-RMV	NODE_19	190	186/189 (98%)
	NODE_41	194	185/194 (95%)
	NODE_155	72	66/69 (96%)
	NODE_159	53	52/53 (98%)
	NODE_231	85	81/82 (99%)
	NODE_252	81	43/44 (98%)

NODE_26

CCGAGAGTTACTCGAACTGGTGTTGTAGGAGCCAGATTTCTGAACTCCCGCCACTCTCTGGGCG
AGCAGGGTCCCATCACTCAAGCACAACACAGAGTTGGATATGCACTTCAACCAACACGCGCGT
AGGCGTCTTGTGAGATCTGTGTTGTTGCGAGTGAGCCGGTTGCGGACTTCCATATCGTCTTGAAG
CATCCATTCCGCAACGCTCCAGTCAAAGCCAGAGCAGTCAGTCGGGACAGTGTGAAGCTGCCA
TTCTTGCAGTAAACGGTCGCTGCTCACTCCACACTGTCTCGCGAGGTTTTGCACGAACTCTCGCG
ATTGGTCATCTGTTGAGAGACCAAAACCGGGTTTCGAGGGTATGGCTCGCCAAAGTGCAATTTC
CCTCTTGTTTTGATTCTGAAACAGAACCCGGGCTACCAGTTGATCCAGCAATGAGACGCTCATG
ATGAGGCGGTAGCGGCCTTCATCCAATTTTGCCT

NODE_38

TTGGGCTTTGATTGCCGCCTCCGGTTGTTCTTTGGGCGTTTCATGGCTGATTCCGCAATTTGTTGA
ACAACCATCCGCTCTATAACTGAGACGTCGATCTTGTTGACCAACGCTTCCACCACCTTGTTGA
GAATATCGCTGTTTGAAGTAGCGACGGGCGAGACTGTAGGGGTGGGCGA

NODE_69
AACCTGTGGGTCCCTCCCAAGGTACTACTTCACAAAACAGAAGGAAGAATCAGAATGGGGGAG
GTTACTTGCAGAGGGAAACCCGGCGCTGGCAGAAAAAGTCAGCGGTTTCGGGTGGCCCCAGTT
CGGTCCCGCAGCCGAA

NODE_111
AGAAACTGGGCAGATTATGAAGACGACATCTCATATGAAGCCCCAAAGTTTCAGGGAAACGAC
GCACGCGGTTCCGTCCGCGGAAACA

NODE_199
GGAAGACTGTGGAGGCATACAAAACTTGTCAAACACAATGCCCCAAGACTTCCCAAAGCAACC
TGCTTGTCTGGGAGAATTTTCT

NODE_19
GAGGATGGCGCATTGATCTTCTGATGAGTCGCGCCAAACTCCACCTCCGATTTGATTGGACGCA
AAGACCCTCCTGCCACCTTTGACGATTGAGAATTTGTTAATGGTGGAGGCAAGGGATGAGAGCT
TGTTGTGTGGATCAAGTTCATAAGCGATGGAACCTTCGGCGGTGGAAGCGGCCTCGGAGATT

NODE_41
AAGACGAAGACGACCAAGTGGAAACACTGCAGGAAGACCTGGAGTCAGACGAGGCTCGCGGG
AGACTTTTGTATTTTCAAAGGACTCTCTCACGGGCAATGCCTCCGGAAAAGTCACCTTCGGGCC
GTCTTTATCAGAGTGTGCAGCATTCAGTGGCGGAATTCTCAAGGCCTACCATGAGTATAAGATC
TCAA

NODE_155
ATTTGCCGCGACAACAACCACTGGCTGGGCCCGGTTATTATTGCGAGCGCGTCGGCGGTTCCTA
GCTCTGCG

NODE_159
ACCTATCTATCTCCTCGAACGTGCGTTCAATTGTGAATGAATACGGGAGGTAG

NODE_231
TTCGGGTTTTGAACATTGACCTCAATCGTGATCCTGAAGGAACCCGCGACTGAGGACTTTCCGTT
ACCTTTGTAGAGGATGGCGC

NODE_252
GAGAACTCCATGAGCAGCTGCCCCGTCTACCTATCTCGGATTTTGAACATTGACCTCAATCGTG
ATCCTGAAGGAACCCGC

Table S5. Percentages of nucleotide substitutions in MaYMV genome.

	Percentage of Nucleotide Substitution (%)			
	**A**	**T**	**C**	**G**
A	-	5.0	1.5	32.7
T	-	-	58.5	1.7
C	-	-	-	0.6
G	-	-	-	-

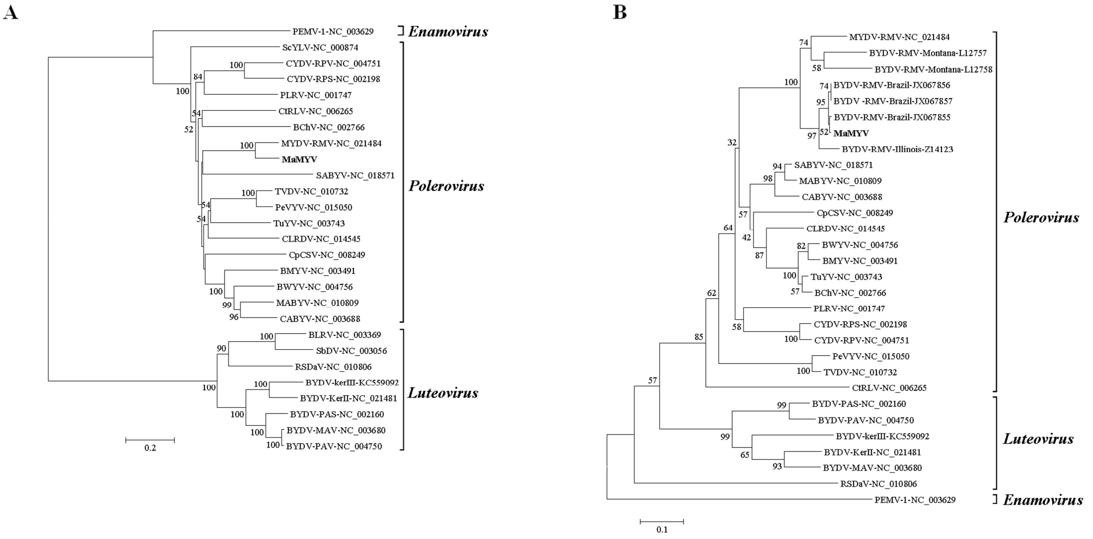

Figure S1. Phylogenetic analyses of MaYMV and selected luteoviruses based on amino acid sequences of RdRp (**A**) or CP (**B**). The phylogenetic trees were generated using the Neighbor-Joining method and MEGA6 software. The percentage of replicate trees in which the associated taxa clustered together in the bootstrap test (1000 replicates) is shown next to the branches.

© 2016 by the authors; licensee MDPI, Basel, Switzerland. This article is an open access article distributed under the terms and conditions of the Creative Commons by Attribution (CC-BY) license (http://creativecommons.org/licenses/by/4.0/).
